# Neuroendocrine carcinoma of the cervix: a systematic review of the literature

**DOI:** 10.1186/s12885-018-4447-x

**Published:** 2018-05-04

**Authors:** Clemens B. Tempfer, Iris Tischoff, Askin Dogan, Ziad Hilal, Beate Schultheis, Peter Kern, Günther A. Rezniczek

**Affiliations:** 1 Department of Obstetrics and Gynecology, Ruhr-Universität Bochum, Bochum, Germany; 2 Department of Pathology, Ruhr-Universität Bochum, Bochum, Germany; 3 Department of Hematology and Oncology, Ruhr-Universität Bochum, Bochum, Germany; 4 Department of Obstetrics and Gynecology, St. Elisabeth Hospital, Bochum, Germany

**Keywords:** Neuroendocrine, Cervical cancer, Small cell cancer, Large cell cancer, Chemotherapy, Radical surgery

## Abstract

**Background:**

Neuroendocrine carcinoma of the cervix (NECC) is a rare variant of cervical cancer. The prognosis of women with NECC is poor and there is no standardized therapy for this type of malignancy based on controlled trials.

**Methods:**

We performed a systematic literature search of the databases PubMed and Cochrane Central Register of Controlled Trials to identify clinical trials describing the management and outcome of women with NECC.

**Results:**

Three thousand five hundred thirty-eight cases of NECC in 112 studies were identified. The pooled proportion of NECC among women with cervical cancer was 2303/163470 (1.41%). Small cell NECC, large cell NECC, and other histological subtypes were identified in 80.4, 12.0, and 7.6% of cases, respectively. Early and late stage disease presentation were evenly distributed with 1463 (50.6%) and 1428 (49.4%) cases, respectively. Tumors expressed synaptophysin (424/538 cases; 79%), neuron-specific enolase (196/285 cases; 69%), chromogranin (323/486 cases; 66%), and CD56 (162/267; 61%). The most common primary treatment was radical surgery combined with chemotherapy either as neoadjuvant or adjuvant chemotherapy, described in 42/48 studies. Radiotherapy-based primary treatment schemes in the form of radiotherapy, radiochemotherapy, or radiotherapy with concomitant or followed by chemotherapy were also commonly used (15/48 studies). There is no standard chemotherapy regimen for NECC, but cisplatin/carboplatin and etoposide (EP) was the most commonly used treatment scheme (24/40 studies). Overall, the prognosis of women with NECC was poor with a mean recurrence-free survival of 16 months and a mean overall survival of 40 months. Immune checkpoint inhibitors and targeted agents were reported as being active in three case reports.

**Conclusion:**

NECC is a rare variant of cervical cancer with a poor prognosis. Multimodality treatment with radical surgery and neoadjuvant/adjuvant chemotherapy with cisplatin and etoposide with or without radiotherapy is the mainstay of treatment for early stage disease while chemotherapy with cisplatin and etoposide or topotecan, paclitaxel, and bevacizumab is appropriate for women with locally advanced or recurrent NECC. Immune checkpoint inhibitors may be beneficial, but controlled evidence for their efficacy is lacking.

**Electronic supplementary material:**

The online version of this article (10.1186/s12885-018-4447-x) contains supplementary material, which is available to authorized users.

## Background

Neuroendocrine neoplasias (NENs) are aggressive malignancies derived from neuroendocrine cells. The term neuroendocrine refers to the fact that the tumor cells originate from the embryonic neuroectoderm and display an immunohistochemical profile consistent with endocrine glandular cells [1]. They may or may not secrete peptide hormones. In humans, NENs are typically located in the gastrointestinal tract, the pancreas, and the lungs and are subdivided in well-differentiated NENs and poorly differentiated NENs [2]. Well-differentiated NENs include neuroendocrine tumors (NET) G1 (also known as typical carcinoid), NET G2 (also known as atypical carcinoid), and NET G3. Poorly differentiated neuroendocrine carcinomas (NECs) include small cell NEC and large cell NEC (Table 1).

**Table 1:** Grading of neuroendocrine neoplasias of the cervix

Classification/Grade	Mitotic Index^a^	Ki-67 Index^b^
Well-differentiated NEN		
NET G1	< 2/10	≤ 2
NET G2	2–20	3–20
NET G3^c^	> 20	> 20
Poorly differentiated NEN		
NEC G3	> 20	> 20
Small cell carcinoma		
Large cell carcinoma		

According to Kim et al. [2]. *NEN* neuroendocrine neoplasia, *NET* neuroendocrine tumor, *NEC* neuroendocrine carcinoma

^a^Mitotic index: based on the evaluation of mitoses in 50 high-power fields (HPF; 0.2 mm^2^ each) in areas of higher density; expressed as mitoses per 10 high-power fields (mitoses/2 mm^2^)

^b^Ki-67 proliferation index: based on the evaluation of ≥500 tumor cells in areas of higher nuclear labeling (so-called hotspots)

^c^NET G3 is defined as NET with Ki-67 proliferation index/mitotic index > 20 and without morphological features of small cell NEC or large cell NEC

Rarely, NENs may also occur in other organs such as the female genital tract [3]. Neuroendocrine carcinoma of the cervix (NECC) is an aggressive histological variant of cervical cancer accounting for about 1–1.5% of all cervical cancers [1, 4]. Small cell NEC is the most common type of NECC, whereas well-differentiated NETs, especially NET G1 (typical carcinoid) and NET G2 (atypical carcinoid), are very rare at this location [5]. Grading of NECC is similar to NEN of other locations like lung or the digestive system (Table 1). Due to the rarity of this malignancy, the management of NECC is difficult and associated with uncertainty. An interdisciplinary approach is necessary, because most studies investigating the treatment of neuroendocrine tumors have been performed in patients with tumors in organs other than the cervix, mostly the lung and pancreas [4, 6]. Specifically, neuroendocrine tumors mainly occur in the lungs, and thus treatment schedules for neuroendocrine tumors originating in other organs are similar to those used in small cell lung cancer. The biology of NECC is different from squamous cell carcinoma or adenocarcinoma of the cervix regarding a number of characteristics. For example, NECC is more likely to invade the lymph-vascular space and to spread to the regional lymph node basin at the time of diagnosis. Also, local and distant relapses occur more often in NECC, and the 5-year overall survival is significantly poorer with around 30% compared to > 65% for squamous cell carcinoma and adenocarcinoma of the cervix [1, 4]. Thus, the aggressive nature of NECC resembles that of small cell lung cancer which, at the time of initial diagnosis, is rarely localized and mostly locally advanced or metastasized.

Positive immunohistochemical staining for neuroendocrine markers like synaptophysin (SYN), chromogranin (CHG), CD56 (N-CAM), and neuron-specific enolase (NSE) is diagnostic for NECC. For establishing the diagnosis, positive staining of at least two neuroendocrine markers is recommended. SYN and CD56 are the most sensitive markers. In some cases of small cell NECC, however, expression of neuroendocrine markers may be negative. Differential diagnosis of NECC includes metastasis of extracervical NEC (e.g. lung or gastro-entero-pancreatic NEC) and extracervical NEC with local wide tumor spread (e.g. urinary bladder, rectum, or Merkel cell carcinoma of the skin). NECC must be distinguished from lymphomas, poorly-differentiated squamous cell carcinomas, and sarcomas or melanomas with morphological small cell-like features. Furthermore, large cell NECC may be positive for p63, a marker strongly expressed in squamous cell carcinomas. In this case, however, positive immunohistochemical staining for neuroendocrine markers excludes the diagnosis of squamous cell carcinoma. While isolated neuroendocrine cells may occur in squamous cell carcinomas and adenocarcinomas, these tumors should not be interpreted as NECs if they lack the morphological features of NECs.

NSE is not only expressed on the surface of NECC tumor cells, but is also present in the serum of the majority of patients and may thus be used as a serum tumor marker for NECC. For example, in a series of six patients with small cell NECC and 13 patients with squamous cell cervical carcinoma, elevated serum levels of NSE were noted in four of six patients with NECC, but in none of the patients with squamous cell carcinoma [7]. Similar to squamous cell cervical carcinoma, high-risk HPV DNA has been detected in the majority of small cell and large cell NECC [8]. In a recent meta-analysis, Castle et al. [9] analyzed HPV infection data in 403 cases of small cell and 45 cases of large cell NECC. They found that 85 and 88% of cases were HPV positive, respectively. The predominant subtypes were HPV18 and HPV16. The authors conclude that HPV infection is the underlying cause for most cases of NECC and that most if not all cases could thus be prevented by prophylactic HPV vaccination.

No treatment schemes for NECC based on prospective clinical trials are currently available due to the rarity of this malignancy. Many authors have therefore used multimodality approaches, mainly derived from the therapy of cervical cancer in general as well as from neuroendocrine tumors of the lung in particular. In 2011, the Society of Gynecologic Oncology (SGO) published a clinical document on the management of women with NECC [10]. They also recommend a multimodality therapeutic strategy. Regarding chemotherapy, the SGO recommends etoposide/platinum-based chemotherapies for NECC but not for well differentiated carcinoid tumors, which should be managed similar to gastroenteropancreatic NETs. The Gynecologic Cancer InterGroup (GCIG), in 2014, also published a consensus review on the treatment of small cell NECC [11]. They recommend radical surgery for early stage disease, either primarily or after neoadjuvant chemotherapy. For patients with advanced stage disease, the GCIG recommends chemoradiation or systemic chemotherapy consisting of etoposide and cisplatin. In line with the SCG and GCIG recommendations, treatment schemes for patients with NECC in the literature usually consist of radical hysterectomy followed by adjuvant chemotherapy for early stage disease. For locally advanced and metastatic disease, definitive concurrent chemoradiation, neoadjuvant chemotherapy followed by surgery, or chemotherapy alone have been described [1, 4]. Various chemotherapy regimens have been reported in women with NECC and they usually differ from those typically used in squamous cell carcinoma and adenocarcinoma of the cervix. For example, Yin et al. used a combination of cisplatin and etoposide in 23 cases of NECC [12]. Other chemotherapy regimens described in the literature are cisplatin/irinotecan [13], carboplatin/paclitaxel [14], and cisplatin/vincristine/bleomycin [15].

To highlight the clinical characteristics, management, and prognosis of women with NECC, we report the results of a systematic review of the literature with cohort studies, case series, and case reports of women with NECC. We discuss the most common therapies and respective outcomes of this malignancy.

## Methods

We performed a systematic literature search of the databases PubMed and Cochrane Central Register of Controlled Trials using the search terms (“neurosecretory systems”[MeSH Terms] OR (“neurosecretory”[All Fields] AND “systems”[All Fields]) OR “neurosecretory systems”[All Fields] OR “neuroendocrine”[All Fields]) AND (“uterine cervical neoplasms”[MeSH Terms] OR (“uterine”[All Fields] AND “cervical”[All Fields] AND “neoplasms”[All Fields]) OR “uterine cervical neoplasms”[All Fields] OR (“cervical”[All Fields] AND “cancer”[All Fields]) OR “cervical cancer”[All Fields]) AND (“therapy”[Subheading] OR “therapy”[All Fields] OR “treatment”[All Fields] OR “therapeutics”[MeSH Terms] OR “therapeutics”[All Fields]). After screening all abstracts of the publications identified by the initial search, studies and case reports reporting on women with NECC were included in the analysis. Suitability of studies was defined for the purpose of this review as reporting on the clinical or biological characteristics, treatment, or clinical outcomes of patients with large cell NECC, small cell NECC, cervical carcinoid tumor, or atypical cervical carcinoid tumor with or without concomitant features of differentiation [16]. In the next step, studies not reporting individual data of women with NECC, duplicate publications, and studies reporting on women with neuroendocrine tumors metastatic to the cervix were excluded. All remaining studies were then retrieved in full and a cross reference search was performed and additional suitable studies reporting on women with NECC as defined above were added to the analysis. Data were extracted, summarized, and analyzed using summary descriptive statistics. Data are given as means or medians where appropriate. No comparative statistics were used.

## Results

**Fig. 1:**
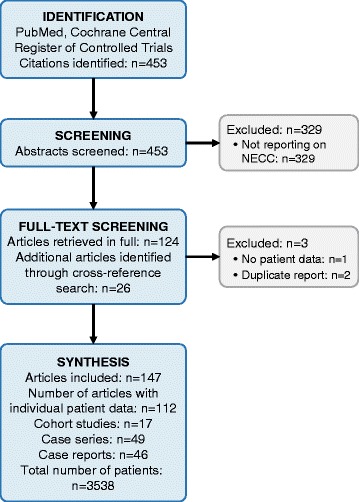
Flow diagram of the literature search algorithm

A systematic literature search of the databases PubMed and Cochrane Central Register of Controlled Trials was performed on 21–10-2017 and identified 453 citations. After screening all abstracts, 124 citations were included in the analysis [1, 7, 8, 12–14, 17–134]. Two studies not reporting individual data of women with NECC, double publications, and a study reporting on women with neuroendocrine tumors metastatic to the cervix were excluded [1, 12, 119]. The 121 selected studies were then retrieved in full and a cross reference search was performed which identified 26 additional studies reporting on women with NECC as defined above [15, 135–159]. Figure 1 shows a flow diagram of the literature search.

We included 147 studies in the final analysis. Table 2 shows study and patient characteristics of 112 studies with individual patient data suitable for pooled analysis. Among these 112 studies, we found 17 retrospective cohort studies, 49 retrospective cases series, and 46 case reports. No prospective studies or interventional trials were identified. Only 8 studies reported on ≥50 patients with NECC describing 130 [59], 100 [100], 68 [71], 64 [129], 61 [142], 57 [134], and 50 [96] cases, respectively. One registry study included 1896 patients without reporting individual patient data [145]. In summary, 3538 cases of NECC have been reported in the literature. Seventeen studies described the total number of cervical cancer patients, among which NECC cases were identified, thus allowing for a calculation of the incidence of NECC among cervical cancer cases. The respective incidences given in these studies were 6/73 (8.22%) [75], 130/2108 (6.17%) [59], 14/389 (3.60%) [108], 10/365 (2.74%) [122], 12/452 (2.65%) [76], 14/649 (2.16%) [103], 44/2835 (1.55%) [109], 1896/127332 (1.49%) [145], 9/677 (1.33%) [56], 31/2385 (1.30%) [48], 25/2201 (1.14%) [120], 11/1370 (0.80%) [117], 64/9474 (0.68%) [129], 14/2074 (0.68%) [150], 6/972 (0.62%) [101], 10/2096 (0.48%) [62], and 7/8018 (0.09%) [123] for a pooled rate of 2303/163470 (1.41%) cases.

**Table 2:** Clinical characteristics of women with neuroendocrine carcinoma of the cervix

Author [Ref]	Year	Study type	# of Cases	Incidence (%)	Early Stage/Advanced stage	Small cell/large cell/other	Immunohistochemical profile (n)	Molecular characteristics (n)
Baggar [135]	2017	CR	1	–	1/0	1/0/0	CHG (1), CD56 (1)	–
Cho [34]	2017	CS	16	–	12/4	16/0/0	SYN (16), CHG (12), ERBB4 (16)	*ATRX* (4/5), *ERBB4* (4/5)
Damian [41]	2017	CR	1	–	1/0	1/0/0	–	–
Kuji [67]	2017	CS	37	–	30/7	29/8/0	SYN (32), CHG (32)	–
Paraghamian [149]	2017	CR	1	–	1/0	1/0/0	–	–
Wu [128]	2017	CR	1	–	1/0	1/0/0	SYN (1), CHG (1)	–
Xie [158]	2017	CS	48	–	40/8	31/1/16	SYN (45), NSE (25), CD56 (13)	–
Cavalcanti [29]	2016	CR	1	–	–	1/0/0	–	p53 (1), MST1R (1), MYCN Ampl. (1), U2AF1 (1), GATA3 (1)
Duan [45]	2016	CS	26	–	22/4	25/0/1	SYN (24), NSE (19), CD56 (13)	–
Frumovitz [47]	2016	COS	44	–	26/18	38/0/6	–	*p53* (5), *PIK3CA* (8), *KRAS* (6)
Ganesan [48]	2016	CS	31	31/2385 (1.30%)	–	31/0/0	–	–
Lee [142]	2016	COS	61	–	41/20	41/7/13	SYN (33/43), CHG (30/43), CD56 (30/43)	–
Margolis [145]	2016	COS	1896	1896/127332 (1.49%)	582/1157	–	–	–
Rajkumar [151]	2016	CR	1	–	1/0	0/1/0	SYN (1), CHG (1), CD56 (1), p16 (1)	–
Robin [100]	2016	COS	100	–	–	–	–	–
Stecklein [153]	2016	COS	40	–	33/7	26/8/6	SYN (36), CHG (26), CD56 (14)	–
Majhi [79]	2015	CR	1	–	1/0	–	SYN (1), CHG (1), NSE (1)	–
Nagao [86]	2015	CS	23	–	18/5	11/12/0	–	–
Sheth [152]	2015	CR	1	–	1/0	1/0/0	SYN (1), CHG (1), CD56 (1)	–
Singh [105]	2015	CR	1	–	1/0	1/0/0	SYN (1), CHG (1)	–
Sodsanrat [109]	2015	CS	44	44/2835 (1.55%)	–	–	–	–
Wang [156]	2015	CR	1	–	1/0	1/0/0	SYN (1), CHG (1), CD56 (1)	–
Wang [157]	2015	CS	32	–	32/0	22/0/10	–	–
Yin [12]	2015	CS	23	–	18/5	23/0/0	–	–
Yuan [132]	2015	CS	38	–	38/0	38/0/0	–	–
Bellefqih [136]	2014	CS	6	–	–	–	–	–
Dongol [139]	2014	CS	4	–	3/1	4/0/0	SYN (4), NSE (4)	–
Lyons [144]	2014	CR	1	–	1/0	1/0/0	–	*KRAS* (1)
Omori [148]	2014	CR	1	–	0/1	0/1/0	SYN (1), CHG (1), CD56 (1), p16 (1), p53 (1)	–
Yin [129]	2014	COS	64	64/9474 (0.68%)	47/17	64/0/0	–	–
Yousef [131]	2014	CR	1	–	0/1	1/0/0	SYN (1)	–
Zaid [134]	2014	CS	57	–	49/8	49/8/0	–	–
Intaraphet [59]	2013	COS	130	130/2108 (6.17%)	82/48	130/0/0	–	–
Kim [63]	2013	CR	1	–	1/0	1/0/0	CHG (1), NSE (1)	–
Kuroda [69]	2013	CR	1	–	1/0	0/1/0	SYN (1), CD56 (1), p16 (1)	–
Li [76]	2013	CS	12	12/452 (2.65%)	8/4	6/6/0	SYN (11), CHG (10), CD56 (11), AE1/3 (12), p16 (11)	–
McCann [146]	2013	COS	26	–	14/12	21/2/2	–	–
Murakami [147]	2013	CR	1	–	0/1	0/1/0	SYN (1), CHG (1), NSE (1)	–
Rekhi [96]	2013	COS	50	–	–	26/14/10	SYN (22/37), CHG (27/37), NSE (7/8), CD56 (8/8)	–
Sitthinumsawan [108]	2013	COS	14	14/389 (3.60%)	9/5	8/3/3	SYN (11), CHG (9), NSE (12), CD56 (11)	–
Wang [125]	2013	CS	13	–	–	13/0/0	SYN (13), CHG (9)	–
Hara [54]	2012	CR	1	–	1/0	0/0/1	SYN (1), CHG (1), CD56 (1)	–
Lin [78]	2012	CS	5	–	4/1	–	–	–
Peng [150]	2012	CS	14	14/2074 (0.68%)	11/3	9/0/5	–	–
Siriaunkgul [106]	2012	CS	26	–	21/5	21/3/2	–	–
Sisti [107]	2012	CR	1	–	1/0	1/0/0	SYN (1), CHG (1), NSE (1)	–
Tanimoto [154]	2012	CR	1	–	0/1	0/1/0	–	–
Teefey [114]	2012	CR	1	–	1/0	1/0/0	SYN (1), CHG (1), CD56 (1), p16 (1)	–
Hao [53]	2011	CR	1	–	1/0	0/1/0	CHG (1), CD56 (1), p16 (1)	–
Komiyama [66]	2011	CR	1	–	1/0	0/0/1	SYN (1), CHG (1), NSE (1)	–
Nasu [13]	2011	CR	1	–	1/0	1/0/0	–	–
McCluggage [84]	2010	CS	21	–	–	13/8/0	SYN (19), CHG (11), CD56 (19), AE1/3 (17), p16 (21), PGP9.5 (9)	–
Niwa [88]	2010	CR	1	–	1/0	0/1/0	–	–
Rashed [95]	2010	CR	1	–	1/0	1/0/0	CHG (1)	–
Bifulco [27]	2009	CR	1	–	0/1	0/1/0	SYN (1), NSE (1)	–
Kajiwara [61]	2009	CS	7	–	2/5	3/2/2	SYN (7), CHG (6)	–
Lee [74]	2009	CR	1	–	1/0	1/0/0	NSE (1), CD56 (1)	–
Li [77]	2009	CR	1	–	0/1	0/1/0	–	–
Wang [123]	2009	CS	7	7/8018 (0.09%)	7/0	0/7/0	–	–
Zivanovic [159]	2009	COS	17	–	11/6	17/0/0	–	–
Albores-Saavedra [21]	2008	CS	2	–	2/0	0/2/0	SYN (2), CHG (2), CD56 (2)	–
Chung [36]	2008	CR	1	–	1/0	1/0/0	SYN (1), CHG (1), CD56 (1)	–
Lee [71]	2008	CS	68	–	68/0	–	–	–
Alphandery [22]	2007	CR	1	–	0/1	1/0/0	SYN (1), CHG (1), CD56 (1), c-Kit (1)	–
Kasamatsu [62]	2007	CS	10	10/2096 (0.48%)	9/1	–	–	–
Ko [64]	2007	CR	1	–	1/0	0/1/0	SYN (1), CHG (1), NSE (1)	–
Tangjitgamol [113]	2007	CR	1	–	0/1	0/1/0	–	–
Cetiner [30]	2006	CR	1	–	1/0	0/1/0	CHG (1), NSE (1)	–
Horn [56]	2006	CS	9	9/677 (1.33%)	–	9/0/0	SYN (7), CHG (7), NSE (7), CD56 (8), p16 (9), p53 (2)	–
Ohwada [89]	2006	CR	1	–	1/0	0/1/0	SYN (1), CHG (1), p16 (1)	–
Wang [124]	2006	CS	31	–	–	25/4/2	–	–
Baykal [26]	2005	CR	1	–	1/0	0/0/1	CHG (1), NSE (1)	–
Chatterjee [32]	2005	CR	1	–	1/0	1/0/0	NSE (1)	–
Tangjitgamol [112]	2005	CS	24	–	16/8	18/6/0	–	–
Tsunoda [117]	2005	CS	11	11/1370 (0.80%)	7/4	11/0/0	SYN (8), CHG (7), NSE (9), CD56 (6)	–
Dikmen [43]	2004	CR	1	–	1/0	0/1/0	SYN (1), CHG (1), NSE (1)	–
Ishida [60]	2004	CS	10	–	–	5/5/0	–	*p53* (1)
Trinh [116]	2004	CR	1	–	1/0	1/0/0	–	–
Viswanathan [121]	2004	COS	21	–	17/4	21/0/0	SYN (19), CHG (16), CD56 (15)	–
Wang [8]	2004	CS	22	–	18/4	22/0/0	SYN (16), CHG (16), NSE (15), p16 (20), Rb loss (18)	–
Hoskins [14]	2003	CS	34	–	17/17	34/0/0	–	–
Sato [101]	2003	CS	6	6/972 (0.62%)	6/0	0/6/0	SYN (6), CHG (6)	–
Conner [39]	2002	CS	23	–	–	18/0/5	SYN (13), CHG (10), p53 (10)	–
Grayson [51]	2002	CS	12	–	–	0/12/0	SYN (11)	–
Marshall [83]	2002	CR	1	–	0/1	–	SYN (1)	–
Ribeiro-Silva [99]	2002	CR	1	–	1/0	0/0/1	CHG (1)	–
Balega [25]	2001	CR	1	–	1/0	–	SYN (1), CHG (1)	–
Bermúdez [15]	2001	COS	20	–	14/6	12/6/2	SYN (8/18), CHG (9/18), NSE (15/18)	–
Boruta [137]	2001	CS	11	–	11/0	9/1/1	–	–
Lenczewski [75]	2001	CS	6	6/73 (8.22%)	–	6/0/0	–	–
Rhemtula [97]	2001	CS	5	–	2/3	0/5/0	SYN (5), MNF116 (5)	–
Straughn [110]	2001	CS	16	–	15/1	15/0/0	SYN (8/13), CHG (8), NSE (12)	*p53* (8), *c-myc* (8/15), *PCNA* (5/15)
Collinet [38]	2000	CS	5	–	2/3	5/0/0	–	–
Delaloge [42]	2000	COS	10	–	7/3	10/0/0	–	–
Herrington [55]	1999	CS	25	–	–	25/0/0	Rb (2)	–
Koch [65]	1999	CR	1	–	1/0	1/0/0	SYN (1), CHG (1)	–
McGarry [85]	1999	CR	1	–	1/0	1/0/0	SYN (1), CHG (1), NSE (1)	–
Wistuba [127]	1999	CS	15	–	–	2/8/5	–	p53 (7), *KRAS* (0), LOH (7)
Yun [133]	1999	CR	1	–	1/0	0/1/0	SYN (1), CHG (1)	–
Mannion [80]	1998	CS	38	–	–	25/5/8	–	LOH (9)
Wang [155]	1998	CS	7	–	7/0	7/0/0	CHG (4), NSE (7)	–
Gilks [50]	1997	CS	12	–	12/0	0/12/0	SYN (6), CHG (12), NSE (4)	–
Toki [115]	1996	CR	1	–	1/0	1/0/0	CHG (1), NSE (1), SER (1)	–
Abulafia [19]	1995	CS	3	–	3/0	3/0/0		–
Abeler [18]	1994	COS	26	–	16/10	26/0/0	SYN (7), CHG (5), NSE (19)	–
Chen [7]	1994	CS	6	–	–	6/0/0	NSE (6)	–
Lewandowski [143]	1993	CS	4	–	2/2	4/0/0	CHG (1), NSE (2)	–
Gersell [49]	1988	COS	15	–	10/5	15/0/0	SYN (10), CHG (11), NSE (11)	–
Sheets [103]	1988	COS	14	14/649 (2.16%)	14/0	14/0/0	–	–
van Nagell [120]	1988	CS	25	25/2201 (1.14%)	–	25/0/0	CHG (8), NSE (8)	–
Walker [122]	1988	CS	14	10/365 (2.74%)	8/6	12/0/2	–	–
Turner [118]	1986	CR	1	–	1/0	1/0/0	–	–
*Pooled Analysis*	*–*	*–*	*3538*	*2303/163470 (1.41%)*	*1479 (50.7%)/ 1436 (49.3%)*	*1123 (80.0%)/ 176 (12.5%)/ 105 (7.5%)*	*SYN (424/538; 79%), CHG (323/486; 66%), NSE (196/285; 69%), CD56 (162/267; 61%), AE1/3 (29/33; 88%), c-Kit (1/1; 100%), ERBB4 (16/16; 100%), MNF116 (5/5; 100%), p16 (67/70; 96%), p53 (13/33; 39%), PGP9.5 (9/21; 43%), Rb (2/25; 8%), Rb loss (18/22; 82%), SER (1/1; 100%)*	*ATRX (4/5; 80%), ERBB4 (4/5; 80%), p53 (22/86; 26%), MST1R (1/1; 100%), MYCN Ampl. (1/1; 100%), U2AF1 (1/1; 100%), GATA3 (1/1; 100%), PIK3CA (8/44; 18%), KRAS (7/60; 12%), c-myc (8/15; 53%), PCNA (5/15; 33%), LOH (16/53; 30%)*

*Abbreviations***:** Study types: *COS* cohort study, *CR* case report, *CS* case series. Immunohistochemical profile: CD56 = N-CAM; *CHG* chromogranin, *MNF116* pan-cytokeratin antibody, *NSE* neuron-specific enolase, *Rb* retinoblastoma protein, *SER* Serotonin, *SYN* synaptophysin. Molecular characteristics, *LOH* loss of heterozygosity; *Ampl* amplified

The most common histological subtype of NECC was small cell NECC. Specifically, small cell NECC, large cell NECC, and other histological subtypes were identified in 80.4, 12.0, and 7.6% of cases, respectively. Early (International Federation of Gynecology and Obstetrics [FIGO] stages I to IIA) and late (FIGO stages IIB to IV) stage disease presentation were evenly distributed with 1463 (50.6%) and 1428 (49.4%) cases, respectively.

The immunohistochemical profiles of NECC demonstrated expression of SYN (424/538 cases; 79%), NSE (196/285 cases; 69%), CHG (323/486 cases; 66%), and CD56 (162/267; 61%) as the most typical markers of NECC. Only a fraction of the published studies analyzed molecular tumor profiles. Among them, the mutations most often identified were in the *p53* (22/86; 26%), *KRAS* (7/60; 12%), *PIK3CA* (8/44; 18%), and *c-myc* (8/15; 53%) genes, respectively. Loss of heterozygosity (LOH) was found to be present in 16/53 (30%) cases. Additional file 1: Figure S1 demonstrates immunohistochemical stainings of a small cell NECC with positive staining for CD56 (N-CAM) and the proliferation marker Ki-67.

Treatment modalities and outcomes are shown in Table 3. The most common primary treatment modality of NECC was radical surgery combined with chemotherapy either as neoadjuvant or adjuvant chemotherapy. Specifically, radical surgery and adjuvant chemotherapy were described in 21/48 studies. Neoadjuvant chemotherapy followed by radical surgery with or without adjuvant therapies (radiotherapy, radiochemotherapy, or chemotherapy) were described in 12/48 studies. Radiotherapy-based primary treatment schemes in the form of radiotherapy, radiochemotherapy with cisplatin, or radiotherapy with concomitant or followed by chemotherapy were also commonly used (15/48 studies). There was no retrospective or prospective comparison of the efficacy of surgery-based, chemotherapy-based, and radiotherapy-based treatment schemes within comparable disease stages in the published studies. After recurrence of NECC, chemotherapy was used in most studies (7/10 studies), followed by radiotherapy (3/10 studies), and surgery (2/10 studies).

**Table 3:** Treatment modalities and outcomes of women with neuroendocrine carcinoma of the cervix

Author	Year	N	Treatment modalities (Initial)	Treatment modalities (Recurrence)	Chemotherapy regimens adjuvant/Neo-Adjuvant	Chemotherapy regimens recurrence/primary advanced	RXT (n)/Gray	Recurrence-free survival duration (m)	Overall survival duration (m)	2- or 3-Year Overall survival rate (%)	5-Year overall survival rate (%)
Paraghamian [149]	2017	1	S + CHXT	CHXT, RXT, NIV	EP	PACTOP	–	1	7	–	–
Wu [128]	2017	1	NACT+S + CHXT	–	IfoEP	–	–	78	78	–	–
Xie [158]	2017	48	NACT+S + CHXT, S + RXT, S + CHXT	–	EP, CPAC, BEP	–	–	–	30	57	31
Lee [142]	2016	61	S + CHXT, RXCT, S + RXCT, CHXT	–	EP, CPAC, FUP	–	–	–	64	–	36
Rajkumar [151]	2016	1	S + CHXT	–	EP	–	–	18	18	–	–
Robin [100]	2016	100	RCXT, NACT+RCXT	–	–	–	–	–	48		
Stecklein [153]	2016	40	S, S + RXT, RXT + CHXT, RCXT	–	–	–	–	9	–	30	27
Nagao [86]	2015	23	S + CHXT, S + RCXT, RXT	–	CPAC, IP	–	–	–	–	64	–
Singh [105]	2015	1	S + CHXT	–	EP	–	–	26	26	–	–
Wang [156]	2015	1	NACT+RXCT	–	EP, N	–	−/25	6	6	–	–
Yin [12]	2015	23	NACT+S + CHXT, NACT+S + RXT + CHXT	–	EP	–	–	–	69 (3), 54 (20)	55	39
Dongol [139]	2014	4	NACT+S + CHXT	–	EP, VPB	–	–	–	10	–	–
Lyons [144]	2014	1	NACT+S + RXCT	MEK-Inh	EP	TRAM	1/45	4	12	–	–
Omori [148]	2014	1	NACT+S + CHXT	–	IP	–	–	21	21	–	–
Kim [63]	2013	1	S + RCXT	CHXT, S	–	CPAC, EP	1/50	4	12	–	–
McCann [146]	2013	26	S, NACT+S, S + RXT	CHXT	EP	EPA	–	34	49	71	–
Murakami [147]	2013	1	CHXT	–	IP	–	–	3	5	–	–
Lin [78]	2012	5	RXT, RXCT, S + RXCT	CHXT	–	FUP, EPD	–	6	22	–	–
Peng [150]	2012	14	S + RXT + CHXT, S + CHXT	–	EP, FUP, EP + CPAC	–	–	6	32	40	25
Teefey [114]	2012	1	NACT+S	–	EP	–	–	24	24	–	–
Komiyama [66]	2011	1	S + CHXT	CHXT, RXT	EPPAC	IP	–	11	19	–	–
Nasu [13]	2011	1	NACT+S + CHXT	CHXT	IP	AMR, EP	–	0	27	–	–
Bifulco [27]	2009	1	S + RXT + CHXT	–	CPAC	–	–	12	12	–	–
Li [77]	2009	1	RXCT	–	EP	–	1/63	21	21	–	–
Wang [123]	2009	7	S, S + CHXT, S + RXT	–	EP, EPEpi, P, PE	–	–	–	32	–	28
Zivanovic [159]	2009	17	S + CHXT, RXCT	–	EP	–	−/14	–	21	30	–
Lee [71]	2008	68	S, NACT+S + RXCT, NACT+S + RXT	–	EP, CPAC, VPB, IPPAC	–	–	–	54	64.6	46.6
Kasamatsu [62]	2007	10	S, S + RXT, S + CHXT	–	EP, PDC	–	2/45–50	8	29	–	43
Ko [64]	2007	1	S + RCXT+CHXT	–	P	–	1/53	24	24	–	–
Tangjitgamol [113]	2007	1	S + CHXT	CHXT	CPAC	CPAC, EP	–	19	27	–	–
Baykal [26]	2005	1	S + CHXT+RCXT	–	EP	–	1/50	21	21	–	–
Tangjitgamol [112]	2005	24	S, S + CHXT, S + RXT, RCXT, RXT, CHXT	–	–	–	–	–	21	–	–
Tsunoda [117]	2005	11	S + CHXT, S + RXT, RCXT	–	EP, PPep	–	3/50; 2/80	–	27	–	27
Trinh [116]	2004	1	S + CHXT+RCXT	–	–	–	–	52	52	–	–
Viswanathan [121]	2004	21	S, RXT, RCXT, S + CHXT, NACT+RXT	–	EP, EPD	–	–	–	41	–	29
Hoskins [14]	2003	34	CHXT+RCXT	–	EP, CPAC	–	–	–	–	60	–
Sato [101]	2003	6	S + RXT + CHXT	–	–	–	–	–	36	–	33
Bermúdez [15]	2001	20	S + RXT, NACT+S, CHXT	–	VBP	–	−/50	–	39	60	39
Boruta [137]	2001	11	S + CHXT	–	VAC, EP	–	–	–	24	38	–
Collinet [38]	2000	5	S + CHXT, S + RXT, RXT		EP, EDC	–	3/55–105	15	17	–	–
Delaloge [42]	2000	3	S + RXT, S + CHXT, S + RXCT	–	PVep	–	–	16	33	–	20
McGarry [85]	1999	1	NACT+RXT	S	EP	–	−/83	0	18	–	–
Abulafia [19]	1995	3	S + CHXT, S + CHXT+RXT	RXT	EVP-16	–	–	30	36	–	–
Lewandowski [143]	1993	4	S + CHXT, CHXT+RXT, NACT+S + RXT, NACT+CHXT	–	EPD	–	2/45	–	25	–	–
Gersell [49]	1988	15	S, RXT, S + RXT, RXT + CHXT	–	*	–	–	–	18	–	33
Sheets [103]	1988	14	S, S + RXT	–	–	–	8/45–60	–	19	15	–
Walker [122]	1988	14	RXT, S, CHXT, S + CHXT, RXT + CHXT	–	VDaCy	–	–	–	14	–	14
Turner [118]	1986	1	S + CHXT+RXT	–	VACyMVP	–	1/45	2	4	–	–
*Pooled Analysis*			S + CHXT (21/48); NACT+S ± Other (12/48); RXT/RCXT or RXT + CHXT (15/43)	CHXT (7/10); RXT (3/10); S (2/10)	EP (24/40); EP + Other (6/40); CPAC (7/40); IP (4/40); FUP (3/40)	EP ± Other (5/8)	–	16 Months	40 Months	50%	34%

*Abbreviations*: *NACT* Neoadjuvant Chemotherapy, *S* Surgery, *RCXT* Radio-Chemotherapy, *E* Etoposide, *P* Cisplatin/Carboplatin, *CHXT* Chemotherapy, *I* Irinotecan, *AMR* Amrubicin, *PAC* Paclitaxel, *Ifo* Ifosfamide, *ICI* Immune-Checkpoint-Inhibitor, *FU* 5-Fluorouracil, *Epi* Epirubicin, *D* Doxorubicin, *C* Cyclophosphamide, *Pep* Peplomycin, *Vep* Vepesid, * Various combinations including Cisplatin, Cytoxan, Methotrexate, Procarbazin, and VP-16¸ VDaCy, Vincristin, Daunorubicin, Cytoxan, *VACMVP* Vincristin, Adriamycin, Cyclophosphamide, Methotrexate, VP-16, *V* Vincristine/Vinblastin, *TOP* Topotecan, *NIV* Nivolumab, *N* Nedaplatin, *MEKInh* MEK-Inhibitor, *TRAM* Trametinib, *A* Adriamycin

There is no standard chemotherapy regimen for NECC, but cisplatin/carboplatin and etoposide (EP) was the most commonly used treatment scheme (24/40 studies), similar to the treatment routinely used for small cell lung cancer. EP combined with other substances such as bleomycin, cyclophosphamide, or doxorubicin was reported in another 6/40 studies, making EP alone or in combination by far the most commonly used cytotoxic regimen. Other commonly used cytotoxic regimens in the primary therapy setting (neoadjuvant or adjuvant) were cisplatin/carboplatin and paclitaxel (7/40 studies) and cisplatin combined with irinotecan (4/40 studies). Other regimes such as 5-fluorouracil/cisplatin, vincristine/cisplatin/bleomycin, vincristine/adriamycin/cisplatin, and irinotecan/cisplatin/paclitaxel were only rarely used. In women with recurrent NECC, EP alone or in combination with other cytotoxic drugs was also the most commonly used cytotoxic regimen (5/8 studies). Overall, the prognosis of women with NECC was poor. The recurrence-free survival was short with a mean duration of 16 months and the mean overall survival duration of women with NECC was 40 months. In a pooled analysis of all studies reporting absolute survival rates, the 2-year- and 5-year overall survival rates were 50 and 34%, respectively.

Targeted therapies and immune-checkpoint inhibitors were only described in three studies [102, 144, 149]. Paraghamian et al. used nivolumab in a patient with recurrent, metastatic, programmed cell death 1 ligand 1 (PD-L1)-negative small cell NECC, who experienced a complete response [149]. Sharabi et al. report a patient with metastatic, chemotherapy-refractory NECC with bowel obstruction due to a large tumor burden [102]. Liquid biopsy demonstrated a high number of tumor mutations. She was treated with radiotherapy combined with nivolumab and experienced a near-complete systemic resolution of disease for at least 10 months. Lastly. Lyons et al. used the mitogen-activated protein kinase 1 (MEK)-inhibitor trametinib in a woman with recurrent small cell NECC and a Kirsten rat sarcoma gene (KRAS)-mutated tumor [144]. This patient also experienced a complete response.

The largest cohort of women with NECC was published by Margolis et al. [145]. Using the National Cancer Database (NCDB), the authors identified 1896 patients with NECC. These patients were younger, more often white, and diagnosed with metastatic disease at presentation compared to women with squamous cell cervical cancer. In a multivariable analysis, NECC patients of all tumor stages had a significantly higher risk of death compared to women with squamous cell cervical cancer. Three other large cohorts analyzed data sets of 188 [37], 130 [59], and 100 [100] cases, respectively. Cohen et al. summarized the characteristics and treatment results of 188 patients most of whom had early stage disease (*n* = 135 with FIGO stages I-IIA) [37]. The 5-year disease-specific survival in FIGO stages I-IIA, IIB-IVA, and IVB disease were 36.8, 9.8, and 0%, respectively. In this patient cohort, adjuvant chemotherapy or chemoradiation was associated with a significantly improved survival in all patients. Consequently, use of chemotherapy or chemoradiation was an independent prognostic factor for improved survival. Robin et al. used the National Cancer Data Base to identify 100 women with locally advanced NECC treated with definitive chemoradiotherapy [100]. There was a substantial improvement in overall survival when brachytherapy was administered in addition to external beam radiotherapy resulting in an improved median survival of 48.6 vs. 21.6 months. Intaraphet et al. looked at 130 patients with small cell NECC and identified older age and locoregional lymph node involvement as the most important prognostic factors among surgically treated patients [59].

The largest series of women analyzing the treatment efficacy of chemotherapy among women with recurrent NECC was published by Frumovitz et al. [140]. They compared 13 patients who received the combination of topotecan, paclitaxel, and bevacizumab (TPB) with 21 patients receiving other regimens, mostly a platinum-based regimen with or without a taxane. TPB was associated with a significantly improved outcome. For example, the median progression-free survival was 7.8 months for TPB and 4.0 months for non-TPB regimens and the median overall survival was 9.7 months for TPB and 9.4 months for the non-TPB regimens. Eight women (62%) who received TPB versus four (19%) who received non-TPB regimens were on treatment for > 6 months, and four patients (31%) in the TPB group versus two (10%) in the non-TPB group were on treatment for > 12 months.

The bulk of studies identified in this systematic review were small case series (43.8%) and case reports (41.1%). As expected, the heterogeneity among these studies with low numbers of NECC patients was considerable. However, as shown in Table 3, most patients were treated with radical surgery and adjuvant or neoadjuvant chemotherapy, whereas chemotherapy alone or radio/chemo/therapy alone were rarely used. Long-term survivors among these women were almost exclusively found in cases with early stage disease at initial presentation, complete tumor resection, and chemotherapy with or without radiotherapy.

## Discussion

NECC is an aggressive histological variant of cervical cancer accounting for 1.4% of all cervical cancers. The management of NECC is difficult and is associated with uncertainty. Therefore, we performed a systematic review of the literature and identified data of 3538 NECC cases from 112 studies. We found that NECC is a rare variant of cervical cancer with small cell NECC being the most common histological subtype. This tumor carries a poor prognosis with a mean overall survival of 40 months and a 5-year overall survival rate of 34%. Multimodality treatment with radical surgery and adjuvant or neoadjuvant chemotherapy with etoposide and cisplatin is the mainstay of treatment for early stage disease while combined radiochemotherapy and chemotherapy are appropriate for women with locally advanced or recurrent NECC. A large number of chemotherapy regimens have been described in the treatment of patients with NECC but cisplatin/carboplatin and etoposide alone or in combination with other substances have been described in more than two thirds of the published studies. Novel therapeutics such as immune checkpoint inhibitors and targeted therapies may be beneficial, but evidence for their efficacy is lacking.

Although there is no standard of care regarding the choice of chemotherapy for women with NECC, we found that cisplatin/carboplatin and etoposide was the most commonly used regimen in the primary treatment and may thus be regarded as an informal standard. Of note, this combination was described in 30/40 studies. The exact dosage and therapy duration of this scheme, however, varied considerably in the published studies. For example, Baykal et al. used cisplatin 80 mg/m^2^ on day 1 together with etoposide 120 mg/m^2^ on days 1, 2, and 3 in a 21 day cycle [26]. Intaraphet et al. used cisplatin 75 mg/m^2^ and etoposide 100 mg/m^2^ every 3 weeks [59]. Hoskins et al. used etoposide (40 mg/m^2^/d) and cisplatin (25 mg/m^2^/d) over 5 consecutive days starting on days 1, 15, 29, and 43 and combined this scheme with locoregional irradiation started on day 15 [14].

In women with recurrent NECC, cisplatin/etoposide alone or in combination with other cytotoxic drugs was also the most commonly used cytotoxic regimen described in 5/8 studies. Of note, women with recurrent disease who had already been treated with cisplatin/carboplatin and etoposide in the primary setting might benefit from a triplet regimen consisting of topotecan, paclitaxel, and bevacizumab. In the largest series of women with recurrent NECC, Frumovitz et al. found that the combination of topotecan, paclitaxel, and bevacizumab was superior to platinum-based regimens with or without a taxane [140]. Thus, in women who already had received cisplatin/carboplatin and etoposide in the primary treatment, topotecan, paclitaxel, and bevacizumab might be an appropriate choice.

Women with NECC have a poor prognosis irrespective of the treatments used. Even with aggressive treatment schemes involving radical surgery, chemotherapy and radiotherapy, the mean 5-year overall survival rate was only 34% in our pooled analysis of the published data. Therefore, new treatment concepts are warranted for this subgroup of cervical cancer patients. Targeted therapies and immune-checkpoint inhibitors might be such new treatment options for NECC. In two case reports, nivolumab led to durable remissions in patients with recurrent disease as did the MEK-inhibitor trametinib in a woman with recurrent small cell NECC and a *KRAS*-mutated tumor [102, 144, 149]. Clearly, this is not a broad evidence base. On the other hand, NECC is a very rare disease and in view of a reasonable alternative, these novel agents might be used in women with recurrent NECC and progression after conventional chemotherapy regimens such as cisplatin/etoposide or topotecan, paclitaxel, and bevacizumab. When comparing these regimens to those usually used for small cell lung cancer, platinum compounds, etoposide, topotecan and anthracyclines are familiar substances whereas paclitaxel or bevacizumab are rarely used in small cell lung cancer.

## Conclusions

We found that NECC is a rare form of cervical cancer with a poor prognosis. Due to the small number of cases and the retrospective nature of this analysis, conclusions are limited, but multimodality treatment with radical surgery and adjuvant or neoadjuvant chemotherapy with etoposide and cisplatin is the mainstay of treatment for early stage disease while combined radiochemotherapy and chemotherapy are appropriate for women with locally advanced or recurrent NECC. In light of the poor prognosis of women with NECC despite aggressive treatment, novel therapeutics such as immune checkpoint inhibitors and targeted agents should be incorporated into the management even without controlled evidence.

## Additional file

Additional file 1: **Figure S1.** Immunohistochemical stainings of a small cell neuroendocrine carcinoma of the cervix. (A) Hematoxylin and eosin staining. (B) Staining for CD56 (N-CAM). (C) Staining for the proliferation marker Ki-67 (using monoclonal antibody MIB-1). Bars, 100 μm. (PDF 1545 kb)

## Abbreviations

CHG: Chromogranin
EP: Cisplatin/carboplatin and etoposide
FIGO: International federation of gynecology and obstetrics
GCIG: Gynecologic Cancer InterGroup
KRAS: Kirsten rat sarcoma gene
LOH: Loss of heterozygosity
MEK-1: Mitogen-activated protein kinase 1
NCDB: National Cancer Database
NEC: Neuroendocrine carcinoma
NECC: Neuroendocrine carcinoma of the cervix
NEN: Neuroendocrine neoplasia
NET: Neuroendocrine tumor
NSE: Neuron-specific enolase
PD-L1: Programmed cell death 1 ligand 1
SGO: Society of Gynecologic Oncology
SYN: Synaptophysin
TPB: Topotecan, paclitaxel, and bevacizumab
