# Supplementary material for: Neuroendocrine carcinoma of the cervix: a systematic review of the literature
Source: BMC Cancer. 2018 May 4;18:530. doi: 10.1186/s12885-018-4447-x (PMC5935948; doi:10.1186/s12885-018-4447-x)
Supplement: Supplementary file 1 — Figure S1. Immunohistochemical stainings of a small cell neuroendocrine carcinoma of the cervix. (A) Hematoxylin and eosin staining. (B) Staining for CD56 (N-CAM). (C) Staining for the proliferation marker Ki-67 (using monoclonal antibody MIB-1). Bars, 100 μm. (PDF 1545 kb) [file 12885_2018_4447_MOESM1_ESM.pdf]

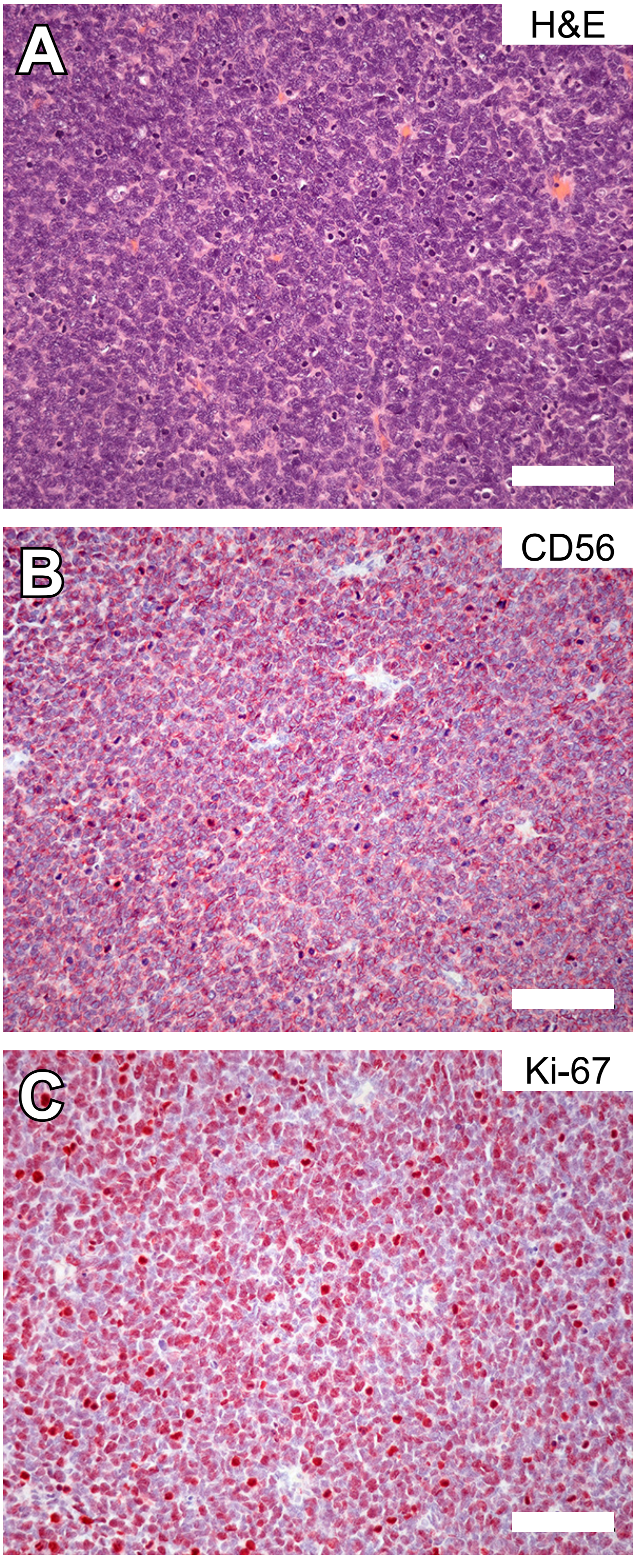

Figure S1. Immunohistochemical stainings of a small cell neuroendocrine carcinoma of the cervix. (A) Hematoxylin and eosin staining. (B) Staining for CD56 (N-CAM). (C) Staining for the proliferation marker Ki-67 (using monoclonal antibody MIB-1). Bars, 100 µm.
